# Caspase-Independent Regulated Necrosis Pathways as Potential Targets in Cancer Management

**DOI:** 10.3389/fonc.2020.616952

**Published:** 2021-02-16

**Authors:** Jianyao Lou, Yunxiang Zhou, Zengyu Feng, Mindi Ma, Yihan Yao, Yali Wang, Yongchuan Deng, Yulian Wu

**Affiliations:** ^1^ Department of General Surgery, The Second Affiliated Hospital, School of Medicine, Zhejiang University, Hangzhou, China; ^2^ Department of Surgical Oncology, The Second Affiliated Hospital, School of Medicine, Zhejiang University, Hangzhou, China; ^3^ Department of General Surgery, Pancreatic Disease Center, Research Institute of Pancreatic Diseases, Ruijin Hospital, Shanghai Jiaotong University School of Medicine, Shanghai, China; ^4^ Department of Nuclear Medicine, The Second Affiliated Hospital, School of Medicine, Zhejiang University, Hangzhou, China

**Keywords:** regulated necrosis, necroptosis, ferroptosis, parthanatos, pyroptosis, cancer therapy

## Abstract

Regulated necrosis is an emerging type of cell death independent of caspase. Recently, with increasing findings of regulated necrosis in the field of biochemistry and genetics, the underlying molecular mechanisms and signaling pathways of regulated necrosis are gradually understood. Nowadays, there are several modes of regulated necrosis that are tightly related to cancer initiation and development, including necroptosis, ferroptosis, parthanatos, pyroptosis, and so on. What’s more, accumulating evidence shows that various compounds can exhibit the anti-cancer effect *via* inducing regulated necrosis in cancer cells, which indicates that caspase-independent regulated necrosis pathways are potential targets in cancer management. In this review, we expand the molecular mechanisms as well as signaling pathways of multiple modes of regulated necrosis. We also elaborate on the roles they play in tumorigenesis and discuss how each of the regulated necrosis pathways could be therapeutically targeted.

## Introduction

As is known to all, the balance between cell survival and cell death determines the homeostasis and growth of organisms. In other words, cell death is an essential natural process of life. Historically, there are two forms of cell death: apoptosis, universally considered to be the regulated and standard cell death form during growth, homeostasis, and pathogenesis (1, 2); and necrosis, mostly considered to be the ‘accidental’ cell death and unregulated cell death form that occurs due to physical or chemical damage (3, 4). Whereas, in the late 1980s, it was suggested for the first time that necrosis might also be genetically regulated. Indeed, Laster et al. reported that the same trigger, tumor necrosis factor (TNF), can cause different forms of cell lysis, either performing nuclear disintegration and “boiling” morphology of cytoplasm (the characteristic of apoptosis) or showing “balloon-like” morphology of cytoplasm without nuclear disintegration (the characteristic of necrosis) (5). Gradually, not only has more and more genetic evidence been confirmed (6–8), but also numerous chemical inhibitors of necrosis have been discovered (7, 9). These findings have indicated the existence of multiple types of regulated cell death (RCD) in addition to caspase-mediated apoptosis. In this context, all of these cell death types are collectively referred to as caspase-independent regulated necrosis.

Cancer is a disease caused by a failure to balance cell division and cell death (10). Nowadays, cancer is the leading cause of death worldwide and causes more than twice the number of deaths in the combination of AIDS (Acquired Immune Deficiency Syndrome), malaria, and tuberculosis (11, 12). Based on World Health Organization statistics in 2016, there were estimated 9.0 million out of 41 million people surveyed died of cancer (13). Although the mortality of dying from cancer between the ages of 30 and 70 had decreased by 19% lower globally between 2000 and 2016, cancer was still the primary cause of premature death, especially in high-income countries (14). Moreover, cancer has caused a substantial social and economic burden to people and eventually remarkably suppressed social and economic development (15, 16). Although this is the case, there is increasing evidence that a considerable proportion of such cancer burden could be avoided with early detection, adequate treatment (17).

Recently, accumulating evidence has demonstrated the involvement of caspase-independent regulated necrosis in cancer pathogenesis (18–22). Furthermore, based on the pathways of caspase-independent regulated necrosis, there are some compounds that have been reported to treat various cancers, such as CD47 agonist peptides, geranylated 4-phenylcoumarins, berberine, and so on (23–25). Therefore, it is necessary to decipher caspase-independent regulated necrosis in cancer conditions because it can provide a brand new view into the pathogenesis of these conditions and contribute to the development of unprecedented targeted anti-cancer treatment.

## Overview of Caspase-Independent Regulated Necrosis

According to morphological differences, RCD can be divided into three distinct forms: apoptosis, autophagy, and regulated necrosis (26). Apoptosis, a best-defined type of RCD, is mediated by caspases, members of the cysteine proteases family. And morphological characteristic of apoptosis includes cell shrinkage, chromatin condensation, and cell disintegration into small fragments (27–30). Autophagic cell death, namely autophagy, is mainly characterized by intracellular autophagosomes (31). Compared with apoptosis and autophagy, regulated necrosis induces cell death independent of caspase activation and lysosome in a genetically controlled manner (32, 33). And regulated necrosis is defined as a type of cell death whose morphological characteristic includes the granulation of cytoplasm and the swelling of organelle and/or cell (4).

Recently, a growing number of research results have enriched people’s understanding of the underlying molecular mechanisms and signaling pathways of caspase-independent regulated necrosis. With accumulating explorations of various modes of regulated necrosis in biochemistry and genetics, the basis of their classification has changed from morphological to more molecular definitions. There are some proper terms to define and classify multiple modes of regulated necrosis (4, 34): necroptosis, ferroptosis, parthanatos, pyroptosis, NETosis, and among others; each of these terms represents a unique concept of cell death process respectively. Although NETosis has been reported to play a role in cancer pathogenesis, NETosis has not been well associated with targeted anti-cancer therapy and will not be discussed in this context.

### Necroptosis

**Figure 1:**
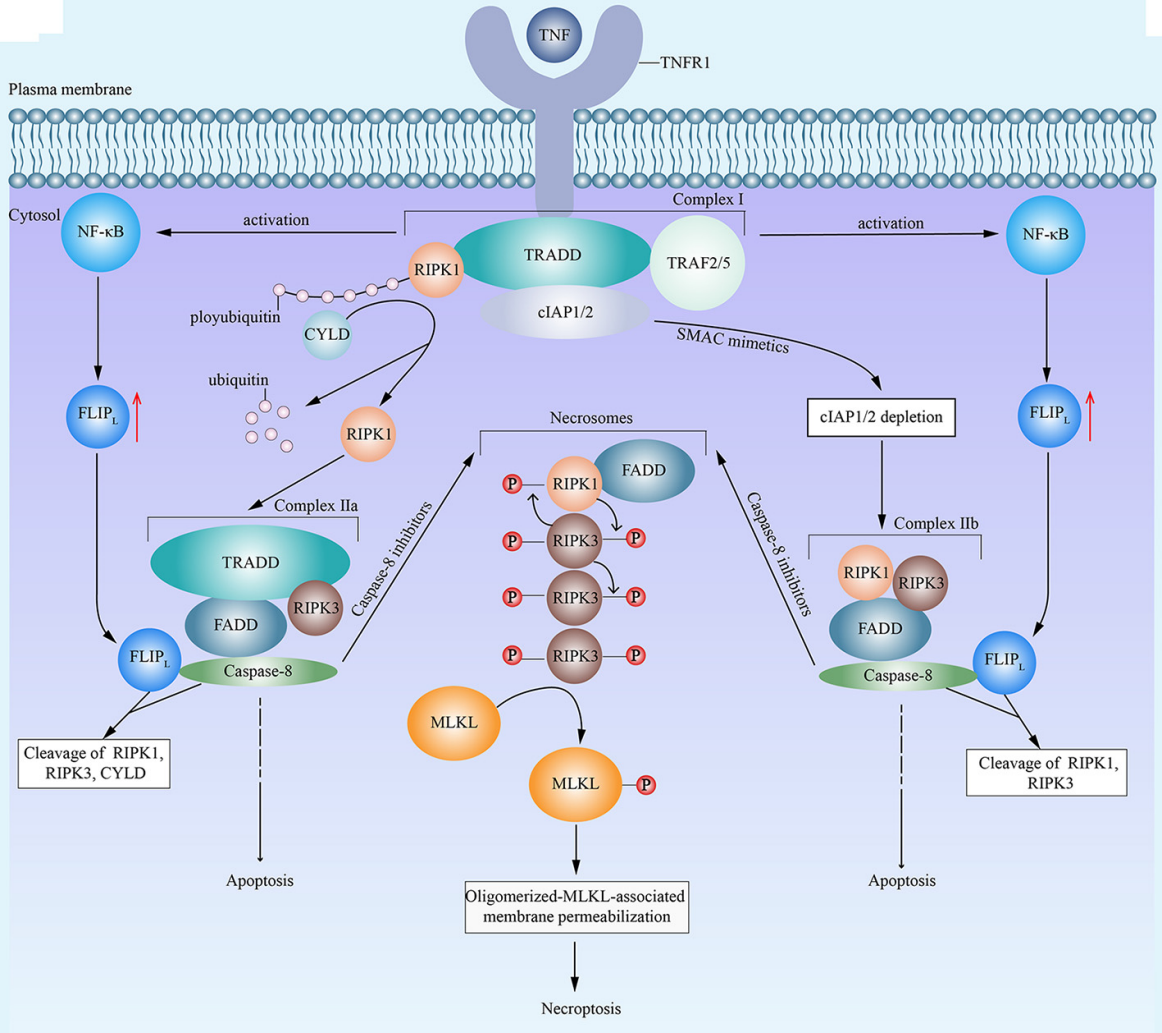
An emerging mode of necroptosis induced by TNF. With the stimulation of tumor necrosis factor (TNF), TNF receptor 1 (TNFR1) recruits TNF-receptor-associated death domain (TRADD). Then, TRADD attracts receptor-interacting protein kinase 1 (RIPK1), cellular inhibitor of apoptosis protein 1 (cIAP1), cIAP2, TNF receptor-associated factor 2 (TRAF2) and TRAF5, and all of above components form the complex I. Complex I then activates the nuclear factor-*κ*B (NF-*κ*B) and promotes the resulting upregulation of FLICE-like inhibitory protein long isoform (FLIP_L_). In complex I, the E3 ligase activity of cIAPs is responsible for the polyubiquitination of RIPK1. On the one hand, cylindromatosis (CYLD) removes the polyubiquitins from RIPK1, which leads to the dissociation of RIPK1 from complex I and the formation of complex IIa. Complex IIa consists of RIPK1, FAS associated death domain (FADD), TRADD, and caspase-8. FLIP_L_ and caspase-8 form a heterodimer to cleave RIPK1, RIPK3 and CYLD, which inhibit the initiation of necroptosis. Then complex IIa leads to caspase-8 homodimerization and activation, resulting in apoptosis. On the other hand, in the presence of second mitochondria-derived activator of caspase (SMAC) mimetics, cIAPs undergo auto-degradation. The depletion of cIAPs facilitates the transition of complex I to complex IIb. Complex IIb contains RIPK1, RIPK3, FADD and caspase-8. Similar to complex IIa, complex IIb leads to apoptosis. However, in the presence of caspase-8 inhibitors, both complex IIa and complex IIb fail to induce apoptosis and turn to cause necroptosis, during which RIPK1 and RIPK3 combine with each other, auto- and trans-phosphorylate each other, and subsequently integrate in necrosomes with FADD. Eventually, phosphorylated RIPK3 recruits and phosphorylates mixed lineage kinase domain-like (MLKL), which leads to the oligomerization of activated MLKL, the translocation of oligomerized MLKL to cell membrane, the permeabilization of MLKL, and consequent necroptosis.

There was a time when people thought that necrotic cell death induced by TNF was negatively regulated by caspase (35). However, this concept has changed by the realization that receptor-interacting protein kinase 1 (RIPK1) (6, 36) and RIPK3 (8, 37) are the key kinases for TNF-induced necrotic cell death (6). Since then, a lot of attention has been attracted to research on regulated necrosis. As further research proceeds, a series of relative molecules have been observed, and regulated necrosis has been proved to be genetically regulated (38, 39). Nowadays, the term necroptosis defines regulated necrosis that is dependent on RIPK1, RIPK3, and mixed lineage kinase domain-like (MLKL) (40). To date, lots of molecules have been identified as triggers of necroptosis, including (but not limited to) TNF (6), CD95L (also known as FASL) (6), TNF-related apoptosis-inducing ligand (6), TNF-related weak inducer of apoptosis (41), CD3/CD28 (42), DNA-dependent activator of IFN-regulatory factors (43), and anti-cancer drugs (such as shikonin) (44). However, it is worth noting that necroptosis is activated under certain circumstances, for instance, when the cells are under severe stress, when some apoptotic signaling pathways are dysfunctional, or when the cells suffer from chemotherapy and they are incapable of performing the apoptotic process (45). When it comes to the signaling pathways, TNF-induced necroptosis is the most representative (**Figure 1**). Under the stimulation of TNF, TNF receptor 1 facilitates the formation of RIPK1- and TNF-receptor-associated death domain (TRADD)-dependent complex I, which is composed of RIPK1, TRADD, cellular inhibitor of apoptosis protein 1 (cIAP1), cIAP2, TNF receptor-associated factor 2 (TRAF2) and TRAF5 (4). Then, complex I leads to the activation of nuclear factor-*κ*B (NF-*κ*B) and resulting upregulation of FLICE-like inhibitory protein long isoform (FLIP_L_) (4). Subsequently, affected by different factors, complex I facilitates the formation of two corresponding different forms of complex II TRADD-dependent complex IIa and RIPK1-dependent complex IIb, respectively (46–48). The deubiquitylation of RIPK1 by cylindromatosis (CYLD) in complex I results in the dissociation of RIPK1 and the formation of complex IIa (46). Besides, the second mitochondria-derived activator of caspase (SMAC) mimetics can representatively cause the auto-degradation of cIAPs (49). And the depletion of cIAPs promotes the transition of complex I to complex IIb (47, 50). Among two forms of complex II, complex IIa consists of RIPK3, FAS associated death domain (FADD), TRADD and caspase-8, while complex IIb contains RIPK1, RIPK3, FADD,and caspase-8 (47). Both complex IIa and complex IIb can induce either apoptosis or necroptosis, which is dependent on whether the caspase-8 activity is present or not, respectively (4, 48). In general, when the activity of caspase-8 is present, FLIP_L_ and caspase-8 combine with each other to form a heterodimer to cleave RIPK1, RIPK3 and CYLD, which prevents the initiation of necroptosis (51). Then, caspase-8 activates a series of downstream singling pathways to execute apoptosis (4). However, under caspase-inhibitory conditions, both of the two forms of complex II induce necroptosis (4), during which RIPK1 and RIPK3 combine with each other and auto- and trans-phosphorylate each other (8, 52). Subsequently, phosphorylated RIPK1, phosphorylated RIPK3, and FADD combine to form microfilament-like complexes, namely necrosomes (50, 53, 54). Then, phosphorylated RIPK3 recruits and phosphorylates MLKL (7, 55). Consequently, oligomerized MLKL binds to the cell membrane and exhibits its permeabilization, which results in cell death (7, 8, 52, 54). Recently, several other events have also been declared to induce necroptosis. It has been reported that RIPK1 phosphorylates signal transducer and activator of transcription 3 (STAT3), which leads to the interaction of STAT3 with gene associated with retinoic and interferon-induced mortality 19 and subsequent translocation of STAT3 to the mitochondria to promote reactive oxygen species (ROS) generation and cell death (56). What’s more, mitochondria may promote the translocation of necrosomes to mitochondria-associated membranes, which probably induces ROS generation and related necroptosis (57).

### Ferroptosis

**Figure 2:**
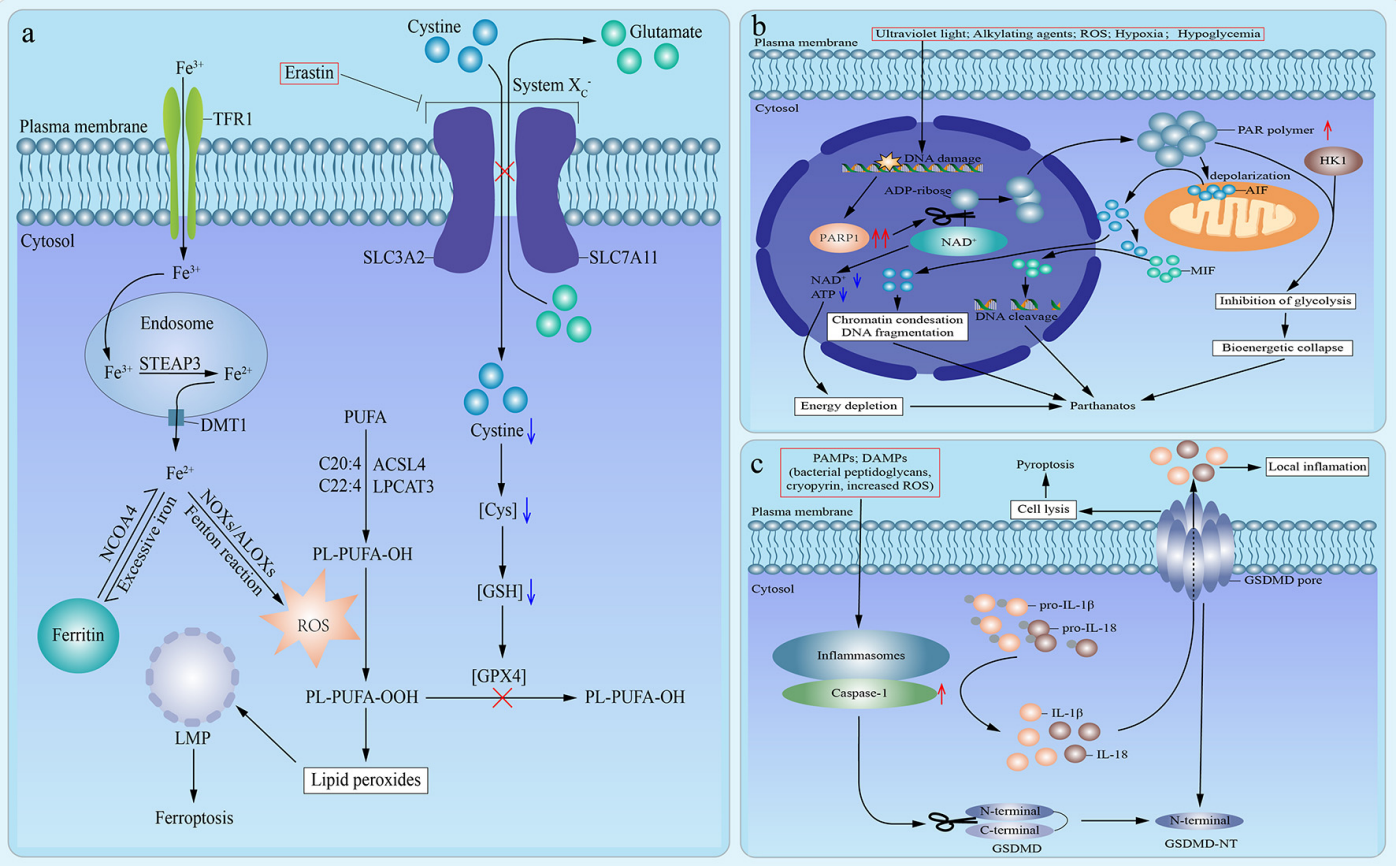
Emerging modes of other types of regulated necrosis. **(A)**. An emerging mode of ferroptosis induced by erastin. In the case of treatment with erastin, the cystine/glutamate antiporter (system $X_{C}^{-}$) is inhibited, which inhibits the exchange of extracellular cystine and intracellular glutamate across cell membrane and results in the lack of intracellular cystine. Then the reduction of cystine to Cys is blocked, which leads to the depletion of glutathione (GSH) and subsequent dysfunction of GSH peroxidase 4 (GPX4). Therefore, GPX4 fails to eliminate lipid peroxides (PL-PUFA-OOH). In the meantime, circulating ferric iron (Fe^3+^) is imported by transferrin receptor 1 (TFR1) and then translocated into the endosome. In the endosome, Fe^3+^ is reduced to ferrous iron (Fe^2+^) by STEAP3. And then Fe^2+^ is released from the endosome into cytoplasm, which is mediated by divalent metal transporter 1 (DMT1). Excess iron is stored in ferritin. And iron is also released from ferritin mediated by cargo receptor NCOA4. Commonly, excessive cytoplastic iron combines with ALOXs and NADPH oxidases (NOXs) to generate reactive oxygen species (ROS) by Fenton reaction. Meanwhile, Free polyunsaturated fatty acid (PUFA) is esterified into cell membrane phospholipids, which is mainly catalyzed by acyl-CoA synthetase long-chain family member 4 (ACSL4) and lysophosphatidylcholine acyltransferase 3 (LPCAT3). Then ROS, which is produced by Fenton-type reactions, oxidizes PUFA-containing phospholipids (PL-PUFA-OH) and converts them to oxidized PUFA-containing phospholipids (PL-PUFA-OOH). And it has been suggested that phospholipids containing arachidonic acid (C20:4) or adrenic acid (C22:4) are the key phospholipids in ferroptosis. Under the simultaneous action of loss-of-function of GPX4 and formation of PL-PUFA-OOH, lipid peroxides accumulate and subsequently facilitate lysosomal membrane permeabilization (LMP), which results in ferroptosis. **(B)** An emerging mode of parthanatos. Under the stimulation of severe DNA damage caused by various factors, poly (ADP-ribose) polymerase 1 (PARP1) is overactivated. The overactivation of PARP1 induces excessive transfer of ADP-ribose groups from oxidized nicotinamide adenine dinucleotide (NAD^+^), which results in the depletion of NAD^+^ and adenosine triphosphate (ATP), subsequent energy depletion, and consequent parthanatos. Moreover, poly (ADP-ribose) (PAR) polymer facilitates the depolarization of mitochondrial outer membrane and the release of active apoptosis-inducing factor ****(AIF) from mitochondria into nucleus, which leads to chromatin condensations and DNA fragmentation, followed by parthanatos. In addition, macrophage migration inhibitory factor (MIF) is promoted to translocate into nucleus by cytosolic AIF and leads to parthanatos *via* inducing DNA cleavage. Moreover, hexokinase 1 (HK1) can combine with PAR polymer to inhibit glycolysis, which causes the bioenergetic collapse and parthanatos. **(C)** An emerging mode of pyroptosis. Under the stimulation of pathogen-associated molecular patterns (PAMPs) or danger-associated molecular patterns (DAMPs), inflammasomes are activated, which leads to the recruitment and activation of caspase-1. On the one hand, activated caspase-1 induces the maturation and release of interleukin (IL)-1β and IL-18. On the other hand, the activated caspase-1 catalyzes the cleavage of gasdermin D (GSDMD) to promote the formation of N-terminal cleavage product (GSDMD-NT), which targets and binds to the selected plasma membrane phosphoinositide. Consequently, the interaction of oligomerized GSDMD-NT and plasma membrane phosphoinositide accelerates the formation of permeability transition pore and the perforation of cell membranes, which results in cell lysis, release of proinflammatory cytokines, and pyroptosis.

The term ferroptosis was firstly defined in an experimental context by using a lethal small molecule, erastin, to treat *Ras*-transformed tumor cells in 2012 (58). Nowadays, ferroptosis is defined as a mode of caspase-independent regulated necrosis that is dramatically characterized by iron-dependent lipid peroxide accumulation (4, 59). So far, in addition to erastin, there are other small molecule ferroptosis inducers, encompassing Ras-selective lethal small molecule 3 (RSL3), RSL5, buthionine sulfoximine, and ferroptosis-inducing agents (FINs) [including DPI family members (DPI2, DPI7, DPI10, DPI12)] (60, 61). Herein, we use the signaling pathway of ferroptosis induced by erastin as an example (**Figure 2A**). Erastin directly inhibits the cystine/glutamate antiporter (system $X_{C}^{-}$), which results in the lack of intracellular cystine (4). And the depletion of cystine blocks the reduction of cystine to Cys, which leads to the corresponding depletion of glutathione (GSH), an essential intracellular antioxidant synthesized from Cys, and subsequent dysfunction of GSH peroxidase 4 (GPX4) (4, 59, 62). Thus, GPX4 fails to eliminate lipid peroxides (oxidized polyunsaturated fatty acid (PUFA)-containing phospholipids) (59, 60). In the meantime, iron metabolism plays a crucial role in the process of ferroptosis (59). Circulating ferric iron (Fe^3+^) is imported by transferrin receptor 1 (TFR1). Then, Fe^3+^ is translocated into the endosome. In the endosome, Fe^3+^ is reduced to ferrous iron (Fe^2+^) by STEAP3. And Fe^2+^ is released from the endosome into the cytoplasm, which is mediated by divalent metal transporter 1. Commonly, excessive cytoplastic iron combines with lipoxygenases ALOXs and NADPH oxidases (NOXs) to execute Fenton-type reactions, which is the fundamental source of ROS needed for ferroptosis (4, 59, 63). Meanwhile, Free PUFA is esterified into cell membrane phospholipids, which is mainly catalyzed by acyl-CoA synthetase long-chain family member 4 (ACSL4) and lysophosphatidylcholine acyltransferase 3 (LPCAT3). And ROS produced by Fenton-type reactions oxidizes PUFA-containing phospholipids and converts them to oxidized PUFA-containing phospholipids (64–67). Under the simultaneous action of loss-of-function of GPX4 and formation of oxidized PUFA-containing phospholipids, lipid peroxides accumulate and facilitate lysosomal membrane permeabilization, which results in cell death (4, 59). Based on the signaling pathway mentioned above, these ferroptosis inducers could be mainly divided into two classes: one is to cause the depletion of GSH by directly inhibiting system $X_{C}^{-}$, and the other is to directly bind to and inhibit GPX4 without reducing GSH. The former class includes erastin, buthionine sulfoximine (BSO), DPI2, RSL5, and the latter class involves RSL3, DPI7, DPI10, DPI12 (60, 61). Moreover, ferroptosis is the exclusive mode of regulated necrosis that can be repressed by iron chelators (such as deferoxamine) (68), lipophilic antioxidants (such as *α*-tocopherol and coenzyme Q10) (69, 70), and depletion of PUFA (59). What’s more, ferroptosis demands the presence of glutamine and products of glutaminolysis (such as *α*-ketoglutarate) (71). Although both glutaminases GLS1 and GLS2 can catalyze glutaminolysis, only glutaminases GLS2, a transcriptional target of the tumor suppressor p53, can catalyze the ferroptosis-related glutaminolysis. And upregulation of GLS2 contributes to p53-dependent ferroptosis (71, 72). In addition, it has been indicated that cysteine can also be synthesized from methionine through transsulfuration in some cells, which leads to these cells’ resistance to system $X_{C}^{-}$ inhibitors (73).

### Parthanatos

Parthanatos is a kind of regulated necrosis initiated by the overactivation of poly (ADP-ribose) polymerase (PARP)1 (34). PARP proteins, such as PARP1, are ADP-ribosyl transferase enzymes that can catalyze the translocation of ADP-ribose groups from oxidized nicotinamide adenine dinucleotide (NAD^+^) to their target proteins and the synthesis of poly (ADP-ribose) (PAR) polymer (4, 74). And PARP1 plays a fundamental role in the repair system of DNA damage and the maintenance of cellular homeostasis (75). There are some conditions that can cause DNA damage and activate PARP1, such as ultraviolet light (76), alkylating agents (76), the Ca^2+^ signaling pathway (77), posttranslational modifications through acetylation (77), ROS (74), hypoxia (78), hypoglycemia (78). In general, when DNA damage is mild, PARP1 is moderately activated and protects cells through facilitating the repair of DNA damage (79). However, when DNA damage is too severe, PARP1 is overactivated, and its overactivation leads to parthanatos (80, 81).

Typically, the signaling pathway of parthanatos is as follows (**Figure 2B**). The overactivation of PARP1 results in the excessive synthesis of PAR polymer and the depletion of NAD^+^ and ensuing adenosine triphosphate (ATP) deficiency, as NAD^+^ is the immediate substrate for PAR polymer synthesis. Then, NAD^+^ and ATP depletion cause energy depletion, which brings about cell death (77, 78, 82). However, the depletion of NAD^+^ and correlated energy depletion have been reported to be unnecessary for the initiation of parthanatos (83), which indicates the existence of other mechanisms. For instance, PAR polymer leads to the depolarization of the mitochondrial outer membrane and the release of active apoptosis-inducing factor (AIF) from the mitochondria into the nucleus, which results in chromatin condensation and large-scale (about 50 kb) DNA fragmentation, followed by regulated necrosis (74, 77, 78, 80, 84–88). Besides, it has been reported that cytosolic AIF promotes the translocation of macrophage migration inhibitory factor (MIF) from the cytoplasm to the nucleus, and nuclear MIF causes DNA cleavage and consequent cell death (89). Moreover, reportedly hexokinase 1 can combine with PAR polymer to inhibit glycolysis, which causes the bioenergetic collapse and subsequent parthanatos (90, 91). Notably, PAR glycohydrolase (PARG) can reverse all of the above processes and protect cells from PAR-mediated parthanatos *via* catalyzing the degradation of PAR, and knockout of PARG can markedly increase the toxicity of PAR and enhance the occurrence of parthanatos (92, 93).

### Pyroptosis

Initially, Cookson and Brennan coined the term pyroptosis to describe a form of caspase-1-dependent RCD partially similar to apoptosis. This concept was initially introduced as the non-classical cell death of macrophages in the case of bacterial infection (94–98). Thus far, a new definition of pyroptosis has been proposed as a type of regulated necrosis that mainly depends on the activation of caspase-1 and the cleavage of gasdermin D (GSDMD) (99). The pathological stimuli that can trigger pyroptosis include bacterial infection (mainly induced by Gram-negative bacteria), heart attack, and cancer progression (34, 97). Morphologically, pyroptosis is characterized by chromatin condensation, cell swelling, cell membrane lysis, and the intracellular proinflammatory molecule release, including interleukin (IL)-1β and IL18 (75, 99–104).

The canonical process of pyroptosis is as follows (**Figure 2C**). Firstly, pyroptosis can be triggered by numerous pathogen-associated molecular patterns (PAMPs) or danger-associated molecular patterns (DAMPs), such as bacterial peptidoglycans (105), cryopyrin (106), ATP (106), gout-associated uric acid crystals (107), viral double-stranded RNA (108), and the increased intracellular ROS level (109). Secondly, these PAMPs and DAMPs activate intracellular inflammasomes, which leads to the recruitment and activation of inflammatory caspase-1 (96, 104). On the one hand, activated caspase-1 induces the maturation of pro-IL-1β and pro-IL-18, and the release of IL-1β and IL-18 (110). On the other hand, the activated caspase-1 catalyzes the proteolytic cleavage of GSDMD and promotes the formation of the N-terminal cleavage product (GSDMD-NT), which activates the pore-forming activity of GSDMD (102, 111–115). Commonly, GSDMD is usually auto-inhibited by the interaction of its C-terminal repressor domain with its N-terminal pore-forming domain with a loop-like structure (111, 114). Thirdly, GSDMD-NT undergoes oligomerization and binds to the selected plasma membrane phosphoinositide (or cardiolipin), which accelerates the formation of permeability transition pore and the perforation of the cell membrane, followed by cell lysis and release of proinflammatory cytokines and cytosolic contents (102, 104, 114–117).

Recent studies have further enriched the content of pyroptosis. In addition to the most common caspase-1, there are many other caspases reported to be involved in the general process of pyroptosis, such as caspase-3, caspase-4, caspase-5, caspase-8, and caspase-11, relying on corresponding initiating stimuli respectively (101, 118, 119). Besides GSDMD, recent evidence has indicated that other members of the gasdermin family also have the same ability to form permeability transition pore and induce pyroptosis, including GSDMA, GSDMB, GSDMC, and GSDME (101, 112, 114, 120). Moreover, GSDME, the most ancient gasdermin, is specifically cleaved by caspase-3, and the corresponding cleavage product (GSDME-NT) induces the release of lactate dehydrogenase and the perforation of the plasma membrane, which converts chemotherapy drugs- or TNF-induced apoptosis to pyroptosis (121, 122).

## The Role of Caspase-Independent Regulated Necrosis in Cancer and Corresponding Cancer Management

Traditionally, cancer therapy, based on the signaling pathways of RCD, mainly relies on the induction of apoptosis. However, the application of apoptosis inducers in clinical practice shows its limitations and is far from satisfactory for cancer treatment. It is well established that cancer cells often exhibit resistance to therapeutic drugs when treated with apoptosis inducers. Recently, accumulating evidence has suggested that caspase-independent regulated necrosis is involved in cancer initiation and development. Fortunately, a large body of studies on regulated necrosis in cancer have been implemented and gradually revealed a series of intrinsic molecular mechanisms and signaling pathways of regulated necrosis in cancer. Based on these considerable research results, people have had a brand-new perspective about regulated necrosis on the initiation and development of cancer and the development of the anti-cancer drug, which provides new ideas for cancer therapy and brings the gospel to cancer patients. Herein, we will introduce the role of various regulated necrosis modes in the initiation and development of cancer and their potential therapeutic value, respectively.

### Necroptosis

Necroptosis plays a vital role in cancer, while controversy remains.

Most studies have shown that the induction of necroptosis in cancer cells has a potential anti-cancer effect (123). For instance, induction of necroptosis could be an approach to overcome cell death resistance against caspase-8-deficient colorectal cancer (CRC) in a xenograft mouse model (124). Interestingly, it has been reported necroptotic factors are significantly reduced in numerous cancers, such as RIPK1 in neck squamous cell carcinoma (125), RIPK3 in leukemia, breast cancer, melanoma, and CRC (126, 127), and MLKL in gastric cancer, cervical squamous cell carcinoma, pancreatic adenocarcinoma and ovarian cancer (33, 128, 129), and CYLD in chronic lymphocytic leukemia, malignant melanoma and non-Hodgkin lymphoma (130–132). Overall, the downregulation of these crucial factors might prevent cancer cells from undergoing necroptosis pathways and promote the survival of cancer cells, which indicates that these necroptosis-related factors would be the barrier to cancer development. What’s more, necroptosis plays a role in cancer immunosurveillance. Recent research has suggested that RIPK3 promotes anti-cancer immune response by regulating natural killer T cells, as RIPK3(−/−) mice show reduced natural killer T cell responses to metastatic tumor cells (133).

However, necroptosis is a double-edged sword in cancer. The necroptosis-recruited immune inflammatory cells can enhance cancer development *via* inducing angiogenesis, promoting cancer cell proliferation, and accelerating cancer metastasis (134, 135). What’s more, tumor cells can induce endothelial cells to undergo necroptosis, which promotes tumor cells to pass the endothelial barrier from blood vessels and consequently enhances metastasis (136). And these metastasis-promoting effect can be reduced by necrostatin-1 (NEC1), a potent inhibitor of necroptosis through blocking the RIPK1 activity, or endothelial-cell-specific deletion of RIPK3 (136). Accordingly, both loss of RIPK3 in the tumor microenvironment and loss of the kinase activity in RIPK1 have been reported to reduce tumor nodules in the lung in murine B16.F10 primary melanoma tumors, which further implies that RIPK3 and RIPK1 are fundamental for cancer growth and metastasis (137). Moreover, necroptosis contributes to establishing the immune-suppressive tumor microenvironment. It has been observed that RIPK1 and RIPK3 highly express in pancreatic ductal adenocarcinoma (PDAC), and they will be further upregulated through treatment with gemcitabine, a chemotherapy drug (138). Further studies have shown that the necroptosis-induced chemokine attractant CXCL1recruits inhibitory macrophages in a RIPK1/RIPK3-dependent manner, which induces the immune-suppressive microenvironment and promotes PDAC progression. And this signaling pathway can be reversed by the deletion of RIPK3 (138). In addition, necroptosis plays a role in promoting cancer growth. RIPK1, RIPK3, and MLKL have been found to promote tumorigenesis in several breast cancer cell lines, and the suppression of these factors can significantly weaken the tumorigenicity and sensitize therapeutic response to radiotherapy (139). And higher phosphorylation level of MLKL leads to shorter survival and poorer prognosis in patients with colon and esophageal cancer (139).

Taken together, caspase-8-mediated necroptosis has exhibited a dual effect, either suppressing cancer or promoting cancer, on cancer development in different tissues. Therefore, it is believed that the underlying mechanism of necroptosis in different tissues is not yet fully clear. One explanation is that this seemingly paradoxical phenomenon may be interpreted by the different objects of necroptosis. When necroptosis directly causes cancer cells to die, caspase-8-mediated necroptosis often exhibits anti-cancer effect. And when necroptosis triggers inflammatory response in cancer microenvironment, it often exhibits cancer-promoting effects (140).

Notably, despite the Janus-faced intrinsic mechanisms of necroptosis in cancer cells, only natural products and reagents that mainly trigger necroptosis have been generated thus far (**Table 1**).

**Table 1:** Summary of compounds targeting necroptosis in related cancers.

Classification	Compounds	Cancers/Cancer cell lines	Mechanisms	References
Natural products	Shikonin	Osteosarcoma; Pancreatic cancer; Triple negative breast cancer cells	RIPK1/RIPK3↑	(141–143)
		Glioma	RIPK1↑	(144)
		Multiple myeloma; Drug-sensitive cancer cell lines (MCF-7 and HEK293)	Unknown	(44, 145)
	Artesunate	RT4 schwannoma cells; Human primary schwannoma cells	MLKL↑	(146)
	Neoalbaconol	Human nasopharyngeal carcinoma cell lines (C666-1 and HK1); Human breast cancer cell line (MX-1); Human gastric cancer cell line (AGS-EBV)	E3 ubiquitin ligases/cIAP1/cIAP2/TRAFs↓ RIPK1↑ ROS/RIPK3↑	(147)
	Staurosporine	Leukemia U937 cell line	RIPK1/MLKL↑	(148)
	Resibufogenin	Colorectal cancer cells	RIPK3↑	(149)
Reagents	SMAC mimetic BV6	Acute myeloid leukemia cells; Pancreatic cancer cells	cIAP1/2↓ RIPK1/MLKL↑	(150, 151)
	DAPE	Malignant pleural mesothelioma cells (NCI-H28)	RIPK1↑	(152)
	BI2536	Androgen-insensitive prostate cancer cells (LNCaP-AI)	PLK1↓	(153)
	Dorsomorphin	Glioma	Unknown	(154)
	Aurora kinase inhibitor CCT137690	Pancreatic ductal adenocarcinoma cancer	RIPK1/RIPK3/MLKL↑	(155)

RIPK1/3, receptor-interacting protein kinase 1/3; MLKL, mixed lineage kinase domain-like; cIAP1/2, cellular inhibitors of apoptosis protein 1/2; TRAFs, TNFα receptor-associated factors; ROS, reactive oxygen species; SMAC, second mitochondrial-derived activator of caspases; DAPE, 1,2-Diarachidonoyl-sn-glycero-3-phosphoethanolamine; PLK1, Polo-like Kinase 1.

#### Natural Products

Shikonin, an effective naphthoquinone derivative extracted from *Lithospermum erythrorhizon*, has been indicated to induce necroptosis in some kind of cancers (141, 156). Shikonin exhibited its anti-tumor effect in osteosarcoma, glioma, pancreatic cancer, triple-negative breast cancer cells, and multiple myeloma in a dose- and time-dependent manner (141–145). It was suggested that shikonin might increase the expression level of RIPK1 in shikonin-treated glioma cells and the expression level of RIPK1/RIPK3 in shikonin-treated osteosarcoma cells and pancreatic cancer cells, which resulted in the initiation of necroptosis and could be rescued by NEC1 (141, 142, 144) What’s more, it has been reported that the anti-cancer drug resistance of drug-sensitive cancer cell lines, including MCF-7 and HEK293, can be overcome, and these cells show the typical morphologic characteristics of necroptosis when treated with shikonin (44).

Artemisinin, an effective sesquiterpene lactone extracted from *Artemisia annua L.*, has been commonly used to treat malaria (145), and recently it has been discovered to exhibit anti-tumor effect (146). It has been revealed that the artemisinin analog artesunate effectively induces necroptosis in RT4 schwannoma cells and human primary schwannoma cells by facilitating MLKL phosphorylation (146).

Neoalbaconol, a kind of natural compound extracted from *Albatrellus confluens*, has been reported to induce necroptosis in some cancer cell lines, such as human nasopharyngeal carcinoma cell lines (C666-1 and HK1), human breast cancer cell line (MX-1), and human gastric cancer cell line (AGS-EBV). In detail, Neoalbaconol may downregulate E3 ubiquitin ligases, cIAP1/2, and TRAFs to eliminate the ubiquitination of RIPK1 and thus induce necroptosis. Besides, Neoalbaconol may also cause necroptosis by boosting RIPK3-mediated ROS production (147).

Staurosporine, an alkaloid extracted from the bacterium *Streptomyces staurosporeus* (157), has been commonly used to induce apoptosis *in vitro* in some cell types. Recently, Staurosporine has been found to trigger RIPK1- and MLKL-dependent necroptosis in leukemia under caspase-compromised conditions (148, 158).

Resibufogenin, a kind of bioactive compound of bufadienolide family extracted from toad venom (159), has been reported to upregulate RIPK3 to induce necroptosis in CRC cells *in vivo*, which significantly suppresses the growth and metastasis of CRC (149).

#### Reagents

In addition to the above natural products, there are many necroptosis-targeted reagents found in cancer treatment research. Reportedly, the small-molecule SMAC mimetic BV6 could antagonize cIAPs to overcome apoptosis resistance by inducing necroptosis upon caspase inhibition and sensitize acute myeloid leukemia (AML) cells to cytarabine-induced cell death, which could be significantly inhibited by NEC1 or MLKL inhibitor necrosulfonamide but not by caspases inhibitor Z-VAD-FMK (150). Moreover, BV6 alone or combined with TNFα can exhibit the same anti-cancer effect in pancreatic cancer cells (151). Besides, 1, 2-Diarachidonoyl-sn-glycero-3-phosphoethanolamine (DAPE) has been found to induce RIPK1-dependent necroptosis in malignant pleural mesothelioma cells, mainly in NCI-H28 cell line, in a concentration (1–100 μM)-dependent manner (152). What’s more, BI2536, an inhibitor of serine/threonine protein kinase Polo-like Kinase 1 (PLK1), has been reported to induce necroptosis in androgen-insensitive prostate cancer cells (LNCaP-AI) to overcome castration-resistance (153). Moreover, a multicentric parallel phase II trial has demonstrated that intravenous BI2536 has limited anti-cancer activity in five different cancer types, including advanced head and neck, breast, and ovarian cancer, soft tissue sarcoma and melanoma (160). In addition, intravenous BI2536 also exhibited modest efficacy in relapsed non-small-cell lung cancer in an open-label, randomized phase II clinical trial (161), while BI2536 failed to inhibit the progression of relapsed small-cell lung cancer (162). And, the intravenous method of administration of BI2536 has been proven to be safe (163). Also, Compound C (also called dorsomorphin) has been found to induce glioma cell death *via* necroptosis (152). Last but not least, aurora kinase inhibitor CCT137690 can indirectly activate RIPK1, RIPK3, and MLKL in PDAC *in vivo*, which leads to the initiation of necroptosis and the inhibition of cancer growth and metastasis. Indeed, aurora kinase A directly inhibits the activation of RIPK1/RIPK3 and indirectly inhibits the activation of RIPK3/MLKL (155). Nowadays, alisertib is a representative oral aurora kinase A inhibitor. Further clinical research has shown that only a small number of patients with advanced prostate cancer, breast cancer, or small-cell lung cancer can benefit from alisertib monotherapy (164, 165). In addition to single-agent alisertib, a combination of alisertib with irinotecan and temozolomide has shown promising response and progression-free survival rates in patients with relapsed or refractory neuroblastoma (166). And a combination of alisertib with cytarabine and idarubicin also exhibits the same anti-cancer efficacy (167).

Overall, the induction of necroptosis in cancer cells by natural products and reagents is considered a promising therapeutic strategy, especially for apoptosis resistance. Further researches show that these natural products and reagents, which induce necroptosis, have been verified to be not injurious to normal cells (168). However, the underlying mechanism of induced necroptosis still needs further exploration of necroptosis-targeted and cancer-guiding drug research.

### Ferroptosis

A considerable body of evidence has shown that ferroptosis links to cancer pathogenesis and anti-cancer therapy (169). Overall, the induction of ferroptosis inhibits the initiation and development of cancer. However, various regulators of ferroptosis, including some newly identified molecules and genes, exhibit different cancer progression influences. Some of them inhibit ferroptosis to protect cancer cells from cell death, such as GPX4, nuclear factor erythroid 2-related factor 2 (NRF2), CD44v. Others, including BAP1 and iron, induce ferroptosis to kill cancer cells. In addition, p53 and spermidine/spermine N1-acetyltransferase (SAT1) exhibit both cancer-promoting and anti-cancer effects. Because of their differences in their roles, we will explain from the perspective of each molecule in this section.

It has been reported that some kinds of cancers strongly depend on the GPX4 during their pathogenesis and development, such as renal cell carcinomas, diffuse large B cell lymphomas, pancreatic cancer, prostate cancer, melanoma, and a subset of triple-negative breast cancer cell lines with glutamine deficiency (60). And a gene expression analysis has revealed that the expression of GPX4 is higher in malignant pancreatic samples than that in normal tissue samples (170). Besides, a high-mesenchymal cell state has been considered as an essential factor of resistance to chemotherapy drugs in various cancer cell lines, such as the epithelial-mesenchymal transition in epithelial-derived carcinomas, treatment-induced neuroendocrine transition in prostate cancer, and TGF*β*-mediated therapy-resistance in melanoma (171). Further study has demonstrated that this therapy-resistant cell state depends on the activity of GPX4 and inhibition of ferroptosis (171). Therefore, GPX4 is a promising target for drug development to eliminate chemotherapy resistance. The recognition that GPX4 plays such an important role in ferroptosis in various cancers suggests that the inhibitors of GPX4 could point the way for cancer treatment.

NRF2 has been found to prevent hepatocellular carcinoma (HCC) cells from ferroptosis and enhance their therapeutic resistance to erastin, sorafenib, and buthionine sulfoximine. It has been revealed that the interaction of p62 and Kelch-like ECH-associated protein 1 (Keap1) prevents the degradation of NRF2 and promotes its nuclear accumulation (172). Accumulation of NRF2 inhibits ferroptosis by promoting the expression of antioxidant proteins, such as quinone oxidoreductase 1 and heme oxygenase-1, and ferritin heavy chain 1, while knockdown of NRF2 enhances ferroptosis in HCC cells (172).

CD44v, a cancer stem cell marker, is associated with tumor progression (173). Further studies have revealed that CD44v can stabilize system $X_{C}^{-}$, which increases GSH levels and inhibits ferroptosis in cancer cells. Therefore, CD44v expression is responsible for the tumor progression and chemoresistance of various types of cancer cells, including pancreatic cancer, bladder cancer, colon cancer, and head and neck squamous cell carcinoma. Thus, CD44v may be a biomarker for cancers that can be effectively treated with system $X_{C}^{-}$ inhibitors (173).

BAP1 has been found to play an essential role in cancer cell death *via* inducing ferroptosis. *BAP1* is a tumor suppressor gene that encodes a nuclear deubiquitinating enzyme, which regulates various cellular signaling pathways in nucleosomes by eliminating histone 2A ubiquitination (174). Recently, it has been found that BAP1 can inhibit the expression of SLC7A11 to suppress the uptake of cystine by deubiquitinating histone 2A on the SLC7A11 promoter (175). Furthermore, both xenograft model studies and human population studies have confirmed that BAP1 shows its anti-cancer effect by inducing ferroptosis (175).

Iron metabolism plays an essential role in the process of ferroptosis as mentioned above. Interestingly, recent studies have indicated that iron is vital for maintaining the tumor microenvironment due to its prooxidant activity, and its homeostasis is perturbed in various types of cancers, such as bladder cancer, breast cancer, CRC, and so on (176–178). It has been found that iron metabolism disorder is associated with tumorigenesis and tumor growth, for instance, the level of dietary iron is proportional to the formation and development of tumor xenografts in rats (177). However, different results have been observed in the population regarding the relationship between the level of iron and tumorigenesis (176). Some results indicate that the level of iron and tumorigenesis are positively correlated in breast cancer, but negatively correlated results and irrelevant results are also observed (177). What’s more, a recent study has indicated that breast cancer stem cells are more sensitive to ferroptosis than non-cancer stem cells because cancer stem cells have a higher level of TFR1 and iron (179).

The activation of p53, a common tumor suppressor gene, can trigger ferroptosis in certain cancer cells (180), as p53 directly inhibits the transcription of SLC7A11, an essential component of the system $X_{C}^{-}$ (181). P53^3KR^, an acetylation-defective mutant, is a representative case because it specifically inhibits the expression of SLC7A11 rather than other target genes related to anti-proliferative and pro-apoptotic activity (180). Moreover, p53^3KR^ fails to trigger cell-cycle arrest, senescence, and apoptosis but retains the tumor suppression function by inducing ferroptosis *in vivo* (180, 182). However, p53 has been found to limit erastin-induced ferroptosis and protect CRC cells from cell death in a transcription-independent manner (183). Further research has indicated that p53 blocks dipeptidyl-peptidase-4 (DPP4) activity by inducing nuclear accumulation of DPP4, which inhibits DPP4-dependent lipid peroxidation and consequent ferroptosis (183). What’s more, it has been reported that the African-restricted polymorphism S47 in the p53 (p53^S47^) could also inhibit ferroptosis *via* protecting the transcription of SLC7A11 from p53 (184). In addition to direct regulation by p53, p53-targeted downstream SAT1 has also been found to be involved in the regulation of ferroptosis (185). SAT1 could inhibit p53-mediated ferroptosis on the condition when it is knocked out (185). However, when the level of SAT1 elevates, it markedly sensitizes cells to ferroptosis (185).

Accumulating evidence has shown that pharmacological regulation of ferroptosis is feasible for anti-cancer therapy. However, most newly identified regulators of ferroptosis mentioned above have not been targeted yet. Here we list numerous cancer treatment types that have been studied, including natural products, various reagents, chemotherapy drugs, immunotherapy, and nanomedicine (**Table 2**).

**Table 2:** Summary of compounds targeting ferroptosis in related cancers.

Classification	Compounds	Cancers/Cancer cell lines	Mechanisms	References
Natural products	Baicalein	Human pancreatic cancer cells (BxPC-3)	Interaction with lipoxygenase pathway	(186)
	Artenimol	Leukemia cells (CCRF-CEM)	Altering iron-related gene mRNA levels	(187)
	Artesunate	K-*Ras*-mutant PDAC cell lines; Leukemia cells	Altering iron-related gene mRNA levels	(187, 188)
	Cotylenin A plus PEITC	Pancreatic cancer cell lines (MIAPaCa-2, PANC-1, gemcitabine-resistant PANC-1)	ROS↑	(189)
Reagents	Erastin and its derivative (piperazine erastin)	Leukemia; Renal cancer cells; N-*Ras*-mutant HT1080 human fibrosarcoma cells; K-*Ras*-mutant Calu-1 non-small cell lung cancer cells	system $X_{C}^{-}$↓ GPX4↓	(60, 190, 191)
	RSL3	Subcutaneous xenograft tumors derived from BJeLR cells	GPX4↓	(60)
	BSO	BJ cells with oncogenic *Ras* mutation	GSH/GPX4↓ ROS↑	(60)
	Sulfasalazine	Triple-negative breast cancer cells; BJeLR cancer cells; HT1080 cancer cells	System $X_{C}^{-}$↓ GPX4↓	(192)
	APR246	Esophageal cancer	GSH↓	(193)
	Lanperisone	Lung cancer	ROS↑	(194)
	Salinomycin	Breast cancer stem cells	Iron in lysosomes↑ Iron in cytoplasm↓ Ferritin degradation↑ ROS↑	(179)
	Statin drugs	HT-1080 fibrosarcoma cells	coenzyme Q10/tRNA isopentenylation/GPX4↓	(171)
Chemotherapy drugs	Sorafenib	Advanced hepatocellular carcinoma; Primary kidney cancer; Radioactive iodine-resistant advanced thyroid carcinoma	System $X_{C}^{-}$↓	(195, 196)
Immunotherapy	Nivolumab	Human fibrosarcoma cell line (HT-1080); Melanoma cell line (A375)	Activated CD8^+^ T cell↑ IFN-*γ*↑ System $X_{C}^{-}$ (SLC3A2 and SLC7A11) ↓	(197)
Nanomedicine	Combination of MCN, DOX and FeCO	Breast cancer	Local DOX↑	(198)
	Co-assembled nanosystem of photosensitizer Ce6 and erastin	Oral tongue squamous cell carcinoma	Intracellular O_2_↑ ROS↑	(199)
	Combination of iron-oxide nanocarriers with cisplatin(IV) prodrugs	Human ovarian carcinoma (A2780)	Cisplatin(IV) prodrugs in tumor cell cytoplasm↑ ROS↑	(200)
	Combination of erastin, rapamycin and iron-abundant ferritin	Breast cancer	GPX4↓ ferritin degradation↑	(201)
	Combination of Fe_3_O_4_ magnetic nanoparticles, leukocyte membranes, TGF-β inhibitor and PD-1 antibody	Non-small cell lung cancer	H_2_O_2_ level↑ Fenton reaction↑ Microenvironment immunogenicity↑	(202)

PDAC, pancreatic ductal adenocarcinoma; PEITC, phenethyl isothiocyanate; ROS, reactive oxygen species; GPX4, glutathione peroxidase 4; RSL3, Ras-selective lethal small molecule 3; BSO, buthionine sulfoximine; GSH, glutathione; IFN-γ, interferon gamma; MCN, mesoporous carbon nanoparticles; DOX, doxorubicin; FeCO, triiron dodecacarbonyl; Ce6, chlorin e6; TGF-β, transforming growth factor-β; PD-1, programmed cell death protein-1.

#### Natural Products

Baicalein, a natural compound extracted from *Scutellaria baicalensis* and *Scutellaria lateriflora*, has been found to inhibit the proliferation of BxPC-3 human pancreatic cancer cells, which is mediated by its interaction with the lipoxygenase pathway (186). However, recent researches have revealed that baicalein can inhibit erastin-induced ferroptosis in PANC1 and BxPc3 cells, and RSL3-induced ferroptosis in acute lymphoblastic leukemia cells *via* suppressing various lipid peroxidation pathways (203, 204). What’s more, baicalein may activate the Keap1/NRF2 pathway mentioned above to inhibit ferroptosis in HCC cells (172). Therefore, whether baicalein can be used for anti-cancer therapy still needs further research.

Artenimol and artesunate are two kinds of derivatives of artemisinin. Artenimol shows anti-cancer activity partly in a ferroptotic cell death manner but not necroptotic in CCRF-CEM leukemia cells (187). Similarly, artesunate-induced ferroptosis has been observed only in K-*Ras*-mutant PDAC cell lines, but not in human pancreatic ductal epithelial cells or wild-type K-*Ras* PDAC cells, which indicates that the anti-cancer activity of artesunate depends on K-*Ras* mutation (188). However, the artesunate has also been found to induce ferroptosis in leukemia cells in a *Ras*-independent manner (205). Moreover, a rigorous randomized, double-blind clinical trial has suggested that oral artesunate has anti-cancer properties in CRC (206). Furthermore, both oral artesunate and self-administered vaginal artesunate inserts have proven to be safe methods of administration in two independent phase I clinical trials (207, 208).What’s more, artenimol, artesunate, and other eight artemisinin derivatives have been identified to kill cancer cells *via* altering iron-related gene mRNA levels (187).

Cotylenin A (CN-A), a kind of fucicoccan-diterpene glycoside isolated from the metabolites of a *Cladosporium* sp., has been considered as a differentiation inducer of myeloid leukemia cell, and the anti-cancer substance in certain cancer cell lines (189, 209). In addition, phenethyl isothiocyanate (PEITC) has been identified as a dietary anti-cancer compound by inducing ROS production (210). Interestingly, it has been demonstrated that a combined treatment of CN-A and PEITC synergistically promotes the generation of ROS and consequently inhibits cell growth of MIAPaCa-2, PANC-1, and gemcitabine-resistant PANC-1 cell lines, while synthetic CN-A derivatives, including ISIR-005, ISIR-042, and fusicoccin J (a CN-A-related natural product), cannot exhibit this synergy with PEITC (189). And this anti-cancer effect can be inhibited by various antioxidants, such as N-acetylcysteine, ferroptosis inhibitors (such as ferrostatin-1), and the lysosomal iron chelator (such as deferoxamine), but not by apoptosis inhibitors or necroptosis inhibitors, which indicates that CN-A plus PEITC induces ferroptosis in pancreatic cancer cells (189).

#### Reagents

As the pioneer of the discovery of ferroptosis, erastin has been reported to trigger ferroptosis in various cancer cells, which mainly depends on the direct regulation of system $X_{C}^{-}$ and indirect regulation of GPX4 (190, 191). Indeed, leukemia cells and renal cancer cells are more sensitive to erastin than other cancer cells (60). And two members of the mammalian family of mitogen-activated protein kinase, including c-Jun NH2-terminal kinase (JNK) and p38, have been identified to be essential for erastin-induced ferroptosis in leukemia cells (205). Moreover, the anti-cancer activity of erastin has been determined to not correlate with *Ras* mutation (60). However, another study has found that erastin selectively targets certain genotypes in *Ras*-mutant cell lines, including H-*Ras*-mutant-engineered cells, N-*Ras*-mutant HT1080 human fibrosarcoma cells, and K-*Ras*-mutant Calu-1 non-small-cell lung cancer cells (190). In addition to working alone, erastin has been reported to promote the anti-cancer effect of common chemotherapy drugs, including temozolomide, cisplatin, cytarabine, and doxorubicin in certain cancer cells (205, 211, 212). Furthermore, derivatives of erastin, such as piperazine erastin, have been found to have the same anti-cancer ability in tumor xenograft models (60).

Compared with erastin, RSL3 has been found to reprograms cancer cell metabolism by regulating GPX4 without modulating system $X_{C}^{-}$, but its downstream signaling pathways are similar to those of erastin (213). And overexpression of GPX4 results in ferroptotic resistance to RSL3 (213). Indeed, RSL3 can prevent tumor growth of subcutaneous xenograft tumors derived from BJeLR cells (60). And it has been demonstrated that RSL3 can enhance the sensitivity of resistant cancer cells to chemotherapeutic drugs in certain types of cancers (214).

Buthionine sulfoximine (BSO), an irreversible inhibitor of *γ*-glutamyl cysteine synthetase, has been found to reduce GPX4 activity by inhibiting GSH synthesis, and subsequently increase ROS levels, which selectively triggers ferroptosis in BJ cells with oncogenic *Ras* mutation (60). Furthermore, in a phase I clinical trial, BSO infusion has been utilized to enhance the activity of melphalan in patients with high-risk neuroblastoma, as melphalan resistance is induced by increased cellular GSH. And the result of the trial has indicated that BSO may promote the treatment effect of melphalan (215).

Sulfasalazine, an efficient drug for various chronic inflammations, has been found to share the same ability and downstream signaling as erastin and BSO in triple-negative breast cancer cells, BJeLR cancer cells, and HT1080 cancer cells (58, 192, 195). As a common anti-inflammatory drug, oral sulfasalazine has been tested to overcome cisplatin resistance in patients with advanced gastric cancer, but no objective response has been observed (216).

APR246, a mutant-p53 reactivator, has been found to directly bind to and deplete GSH in oesophageal cancer cells, which results in the accumulation of lipid peroxidation and consequent ferroptosis (193).

Lanperisone, which is commonly used as a muscle relaxant, can induce ROS generation to selectively kill K-*Ras*-mutant mouse embryonic fibroblasts *via* ferroptosis signaling *in vitro*, and it can also inhibit tumor growth through inducing ferroptosis in a K-*Ras*-driven mouse model of lung cancer *in vivo* (194, 217). However, the underlying mechanism of lanperisone-induced ROS generation is still unknown.

Salinomycin, a selective agent against cancer stem cells, is a potent ferroptosis inducer in breast cancer stem cells *in vivo* and *in vitro* (179). Salinomycin promotes the accumulation of iron in lysosomes, which subsequently leads to cytoplasmic depletion of iron, degradation of ferritin, and further iron loading in lysosomes. As a result, iron loading causes the excessive generation of ROS *via* Fenton reaction, which enhances lysosomal membrane permeabilization and ferroptosis.

Statin drugs, inhibitors of HMG CoA reductase, may selectively induce ferroptosis in high-mesenchymal state cancer cells (171). Recent studies have revealed that statin drugs sensitize cells to ferroptosis by causing the depletion of coenzyme Q10 and the inhibition of downstream tRNA isopentenylation, which is required for the biosynthesis of GPX4 (70, 171, 218). However, a prospective cohort study has indicated that the use of statin drugs is not associated with the risk of prostate cancer (219). This result may be interpreted by the fact that prostate cancer is not in a high-mesenchymal state.

#### Chemotherapy Drugs

The multikinase inhibitor sorafenib has been reported to directly inhibit System $X_{C}^{-}$ rather than GPX4 activity to induce ferroptosis in advanced HCC similar to erastin (196). In addition to advanced HCC, sorafenib can also cause ferroptosis *via* blocking system $X_{C}^{-}$ in other cancers, including primary kidney cancer and radioactive iodine-resistant advanced thyroid carcinoma (195). The cytotoxic effects of sorafenib can be blocked by pharmacological inhibitors (ferrostatin-1), iron chelator deferoxamine, and NRF2 (172, 196).

#### Immunotherapy

Ferroptosis is involved in cancer immunotherapy, for instance, during nivolumab therapy (197). It has been demonstrated that immunotherapy-induced activated CD8^+^ T cells could downregulate the expression of two essential subunits of system $X_{C}^{-}$, SLC3A2, and SLC7A11, to inhibit the uptake of cystine *via* releasing interferon-gamma (IFN-*γ*), which promotes the accumulation of lipid peroxidation and consequent ferroptosis in cancer cells. In turn, artificial depletion of cystine or cysteine by cyst(e)inase can enhance the anti-cancer efficiency of cancer immunotherapy in mice models. Furthermore, system $X_{C}^{-}$ expression has been found to be negatively associated with activated CD8^+^ T cell level, IFN-*γ* expression, and patient outcome in cancer patients.

#### Nanomedicine

In addition to the above various compounds, a brand-new mode of drug delivery, called nanoparticulate drug delivery system (nano-DDS), has been created to achieve more efficient anti-cancer treatment. Thus far, the application of nano-DDS in ferroptosis has been studied much more than that in other modes of regulated necrosis, so we mainly explain nano-DDS in this section. Compared with traditional administration methods, nano-DDS has the advantages of promoted drug availability and targeting ability, high solubility, and enhanced permeability and retention effect, especially in early-stage solid tumors (220, 221). There are two representative nanomedicines that have been applied in cancer management in clinical practice, involving Doxil and Abraxane (222). And recent studies have shown that ferroptosis-driven nanotherapeutics is a promising anti-cancer therapy strategy because the combination of ferroptosis with bionanotechnology can facilitate targeted delivery of ferroptosis inducers or promoters into cancer cells (220). Indeed, the combination of various therapeutic agents or approaches has been demonstrated to significantly promote anti-cancer efficiency in clinical cancer treatments (223). So far, there are numerous cancer therapeutic combos based on ferroptosis and nano-DDS that have been proven to kill cancer cells in animal models, and these therapeutic combos and their corresponding pharmacological effects are briefly listed in **Table 2**. A more detailed and comprehensive introduction to ferroptosis-driven nanotherapeutics for cancer treatment has been well reviewed (220).

In short, the anti-cancer therapy based on ferroptosis has been well studied until now, and drug researches based on ferroptosis pathways and regulatory factors have gradually emerged. Nowadays, most therapies achieve the effect of eliminating cancer cells by directly or indirectly inhibiting GPX4 and system $X_{C}^{-}$ or promoting ROS generation. These drug studies provide new ideas for cancer treatment, but there is still a long way to go before patients’ actual benefit. Therefore, it is necessary to continue to study related mechanisms and drugs to develop drugs with better curative effects, fewer side effects, and more resistance to drug resistance.

### Parthanatos

The role of PARP1 in cancer management seems to be much more associated with its ability to mediate DNA repair than its ability to induce parthanatos. Indeed, inhibition of PARP1 can induce accumulation of unrepaired single-strand DNA breaks, which results in the collapse of replication forks and consequent generation of double-strand DNA breaks (DSBs) (224–226). Normally, BRCA1 and BRCA2, downstream molecules of PARP1, can execute homologous recombination, an error-free form of DSB repair, which leads to the repair of DSBs (227). When inhibitions of PARP1 and BRCA1/2 coexist, the persistent DNA breaks can induce cell cycle arrest and apoptosis (227). Indeed, increased expression of PARP1 has been found to be a strategy for tumor cells treated by radiation and chemotherapeutic drugs to avoid apoptosis induced by DNA damage, which results in apoptosis resistance (228, 229). In addition, as angiogenesis is a fundamental characteristic of carcinogenesis, it has been found that PARP1 regulates tumor-related gene expression involved in angiogenesis in skin carcinogenesis, such as *HIF-1 α*, *Pecam-1*, and *OPN* (230). And PARP1 inhibition-induced inhibition of *HIF-1 α* might contribute to cancer cell death (231).

Parthanatos is also considered a promising strategy to kill cancer cells when it comes to cancer treatment. Interestingly, unlike other modes of regulated necrosis, both inducers and inhibitors of parthanatos have been found to play an important role in the management of cancer. One of the views is that inhibitors of parthanatos, such as PARP1 inhibitors, could facilitate cancer cell death *via* inhibiting the DNA repair needed for cell survival, while other ideas are that inducers of parthanatos could directly promote cancer cell death (74) (**Table 3**).

**Table 3:** Summary of compounds targeting parthanatos in related cancers.

Classification	Compounds	Cancers/Cancer cell lines	Mechanisms	References
PARP1 inhibitors	3-AB; ISQ; NU1025; AG14361	BRCA1/2-deficient breast cancer cells	Inhibiting DNA repair	(224)
PARP1 inducers	DPT	Glioma cell lines	PARP-1/PAR polymer/AIF↑	(232)
	YM155	Esophageal carcinoma cell lines (KYSE410 and KYSE150)	PARP-1/AIF↑	(233)

3-AB, 3-aminobenzamide; ISQ, 1,5-dihydroxyisoquinoline; NU1025, 8-hydroxy-2-methylquinazolinone; AG14361, 1-(4-dimethylaminomethyl-phenyl)-8,9-dihydro-7H-2,7,9a-benzo[cd]azulen-6-one; DPT, deoxypodophyllotoxin; PARP, poly (ADP-ribose) polymerase; PAR, poly (ADP-ribose); AIF, apoptosis-inducing factor; YM155, sepantronium bromide.

#### PARP1 Inhibitors

It has been found that inhibition of PARP1 and deficiency of BRCA1/2 synergistically kill BRCA1/2-deficient breast and other cancer cells *via* inhibiting DNA repair (224, 225, 234). So far, numerous PARP1 inhibitors have been experimentally utilized for BRCA1/2-deficient cancer treatment, such as 3-aminobenzamide (3-AB), 1,5-dihydroxyisoquinoline (ISQ), 8-hydroxy-2-methylquinazolinone (NU1025) or 1-(4-dimethylaminomethyl-phenyl)-8,9-dihydro-7H-2,7,9a-benzo[cd]azulen-6-one (AG14361) (224). What’s more, as it is mentioned above that PARP1 can protect tumor cells from radiation and chemotherapeutic drugs, PARP1 inhibitors, such as 3-AB, GPI 15427, and nicotinamide, have been utilized as radiosensitizers or chemosensitizer (235–237).

#### PARP1 Inducers

Deoxypodophyllotoxin (DPT), a natural chemical, has been reported to induce parthanatos in glioma cell lines and mice models of xenograft glioma, which can be inhibited by antioxidant N-acetyl-L-cysteine (NAC) and PARP1 inhibitor 3AB. DPT-treated glioma cells demonstrated characteristics that fully comply with those of parthanatos, with the upregulation of PARP1, cytoplasmic accumulation of PAR polymer, and nuclear translocation of AIF (232).

Sepantronium bromide (YM155), a survivin suppressant, has been found to induce parthanatos in cultured KYSE410 and KYSE150 esophageal carcinoma cell lines *in vitro* and relatedly inhibit esophageal squamous-cell carcinoma growth in mice, which can be abrogated by genetic knockdown of PARP1 or AIF (233). Furthermore, sepantronium bromide in combination with rituximab has been suggested to have anti-cancer efficacy in patients with relapsed aggressive B-cell non-Hodgkin lymphoma (238).

In general, researches regarding the signaling pathway of parthanatos are more biased towards neurodegenerative lesions and nerve damage after ischemia. So far, the molecule mechanism of parthanatos has not been widely studied in cancer treatment, and we need to pay more attention to this field for further anti-cancer treatment.

### Pyroptosis

Pyroptosis is mainly observed in macrophages as a vital part of the antibacterial immune defense, but accumulating evidence shows that pyroptosis plays an essential role in some cancer without any bacterial infection, and it is promising to develop an anti-cancer treatment based on pyroptosis (95). Overall, the induction of pyroptosis inhibits the initiation and development of cancer. As GSDMD and GSDME are crucial executors of pyroptosis, here we explain the anti-cancer effects of pyroptosis by introducing the role of GSDMD and GSDME.

GSDMD exerts an essential but distinct role in different cancers, and it exhibits anti-cancer effects. It has been observed that GSDMD is downregulated in gastric cancer, which significantly promotes the proliferation of cancer cells *in vivo* and *in vitro* by enhancing extracellular signal-regulated kinase, STAT3, and phosphatidylinositol 3 kinase/protein kinase B signaling pathways, and regulating cell cycle-related proteins (239).

GSDME, another pyroptosis inducer, also has a substantial impact on the regulation of carcinogenesis. As mentioned earlier, activation of GSDME by caspase-3 can induce pyroptosis. In fact, the expression of GSDME is commonly inhibited due to DNA methyltransferase in most cancer cells (101, 240). Indeed, cleavage of GSDME by caspase-3 has been observed only in certain GSDME-expressing cancer cells, such as SH-SY5Y neuroblastoma cells and MeWo cells (101). Furthermore, experiments concerning human primary cells and GSDME^−/−^ mice indicate that GSDME and its cleavage play a fundamental role in the pyroptosis induced by chemotherapy drugs (101).

Correspondingly, numerous agents and drugs yielding antitumor effects by (at least partially) inducing the activation of GSDMD or GSDME have been proposed hitherto. Moreover, the combination of pyroptosis-targeted therapy and conventional remedies seems to enhance therapeutic efficacy, reduce off-target toxicity, and promote patient outcomes (**Table 4**).

**Table 4:** Summary of compounds targeting pyroptosis in related cancers.

Classification	Compounds	Cancers/Cancer cell lines	Mechanisms	References
Natural products	Galangin	Glioblastoma	Caspase-3/GSDME↑	(241)
	Anthocyanin	Oral squamous cell carcinoma	NLRP3/Caspase-1/IL-1β↑	(242)
	Dioscin	Osteosarcoma	Caspase-3/GSDME↑	(243)
	Berberine	Hepatocellular carcinoma	Caspase-1/GSDMD↑	(244)
	Huaier extract	Non-small-cell lung cancer	Caspase-1/GSDMD↑	(245)
	Curcumin	Malignant mesothelioma cells	Caspase-1/GSDMD/HMGB1↑	(246)
Reagents	L61H10	Lung cancer cell lines	Caspase-3/GSDME↑	(247)
	Metformin	Esophageal squamous cell carcinoma	miR497/PELP1/GSDMD↑	(248)
	DHA	Breast cancer cells	Caspase-1/GSDMD/HMGB1↑	(249)
	DPP8/9 inhibitors	Human acute myeloid leukemia cell lines	Caspase-1/GSDMD↑	(250)
	α-NETA	Epithelial ovarian cancer cells	Caspase-4/GSDMD↑	(251)
	Iron	Melanoma	ROS/GSDME↑	(252)
Chemotherapy drugs	Doxorubicin; Actinomycin-D; Bleomycin; Topotecan; Paclitaxel; Cisplatin	Lung cancer	Caspase-3/GSDME↑	(101, 253)
	Doxorubicin	Melanoma	Caspase-3/GSDME↑	(254)
	5-fluorouracil	Gastric cancer cell lines (SGC-7901 and MKN-45)	Caspase-3/GSDME↑	(255)
	Topotecan; Etoposide; Cisplatin	SH-SY5Y neuroblastoma cells; MeWo cells	Caspase-3/GSDME↑	(101)
	Lobaplatin	Colon cancer cells	Caspase-3/GSDME↑ ROS/JNK/Bax/cytochrome c↑	(256)
Chemo sensitizer	BI2536	Esophageal squamous cell carcinoma cells	Caspase-3/GSDME↑ Inhibiting DNA damage repair	(257)
Molecular targeted therapy	Trametinib; Erlotinib; Ceritinib	K-*Ras*-, EGFR-, or ALK-driven lung cancer	Caspase-3/GSDME↑	(258)
	Combinations of BRAF inhibitors and MEK inhibitors	Melanoma	GSDME/HMGB1↑	(259)
Nanomedicine	As_2_O_3_-NPs	Hepatocellular carcinoma	GSDME↑	(260)

GSDME, gasdermin E; NLRP3, nucleotide-binding oligomerization domain-like receptor pyrin domain containing 3; IL, interleukin; GSDMD, gasdermin D; HMGB1, high-mobility group box 1; PELP1, proline-, glutamic acid- and leucine-rich protein-1; DHA, docosahexaenoic acid; DPP8/9, dipeptidyl peptidase 8/9; α-NETA, 2-(anaphthoyl)ethyltrimethylammonium iodide; ROS, reactive oxygen species; JNK, c-Jun NH2-terminal kinase; Bax, BCL-2 associated X; EGFR, epidermal growth factor receptor; ALK, anaplastic lymphoma kinase; BRAF, B-Raf proto-oncogene; MEK, mitogen-activated protein kinase; As_2_O_3_-NPs, arsenic trioxide nanoparticles.

#### Natural Products

Galangin and anthocyanin, two members of natural pigment belonging to flavonoids, have been reported to promote the cell death of glioblastoma cells and oral squamous cell carcinoma cells, respectively (241, 242). Further studies have revealed that galangin can induce pyroptosis through the caspase-3/GSDME pathway in glioblastoma cells (241). And anthocyanin can kill cancer cells and inhibit the migration and invasion abilities of these cells by inducing pyroptosis, which can be suppressed by caspase-1 inhibitors (242). What’s more, the activated pyroptosis by anthocyanin is associated with increased expression of NLRP3, caspase-1, and IL-1β (242).

Dioscin, a steroidal saponin extracted from *Polygonatum zanlanscianense*, *Dioscorea nipponica Makino*, and *Dioscorea zingiberensis Wright*, has been observed to inhibit the growth of osteosarcoma cells *in vivo* and *in vitro* by inducing pyroptosis (243). Furthermore, mechanistic studies have revealed that dioscin can induce pyroptosis through the caspase-3/GSDME pathway (243).

Berberine, a natural isoquinoline alkaloid with antimicrobial ability, has been found to inhibit the growth, migration, and invasion capacity of HCC *in vivo* and *in vitro* by inducing caspase-1/GSDMD-dependent pyroptosis (244).

Huaier extract, a type of fungus from *Trametes robiniophila*, has shown its anti-cancer ability in non-small-cell lung cancer through caspase-1/GSDMD-dependent pyroptosis *in vitro* and *in vivo* (245). Moreover, a multicentre, randomised clinical trial has indicated that patients with HCC after curative liver resection may benefit a lot from oral Huaier granule (261).

Curcumin, a natural polyphenol extracted from *Turmeric*, has the potential to activate caspase-1 to induce pyroptosis and simultaneously increase the release of proinflammatory factor, high-mobility group box 1 (HMGB1), without processing of pro-IL-1β and pro-IL-18 in malignant mesothelioma cells, which can be inhibited by caspase-1 inhibitor or antioxidant NAC (246). Interestingly, curcumin-induced pyroptosis protects these cancer cells against inflammation (246).

#### Reagents

Compound L61H10, a heterocyclic ketone derivative, has been observed to kill cancer cells without apparent side effects in lung cancer cell lines and mice bearing lung cancer xenografts by inducing pyroptosis (247). Further studies have revealed that compound L61H10 can arrest the cell cycle in the G2/M phase and switch apoptosis to caspase-3/GSDME-mediated pyroptosis (247).

Metformin is a common hypoglycemic agent, and it has been found to induce GSDMD-mediated pyroptosis in esophageal squamous cell carcinoma, a kind of chemo-refractory cancer, *in vivo* and *in vitro* (248). Further studies have revealed that metformin can trigger pyroptosis *via* targeting miR497/Proline-, glutamic acid- and leucine-rich protein-1 axis, which indicates a new therapeutic target for treating esophageal squamous cell carcinoma (248). In addition, a phase 1 dose-finding study of metformin has demonstrated that metformin can promote the efficiency of chemoradiotherapy and elevate the rates of overall survival and progression-free survival in patients with locally advanced head and neck squamous cell carcinoma (262). Similarly, metformin plays an active anti-cancer effect in breast cancer patients (263).

Docosahexaenoic acid (DHA), a kind of omega-3 fatty acid, has been considered a pyroptosis inducer in breast cancer cells (249). Mechanistic studies have revealed that DHA can inhibit the growth of breast cancer cells and kill cancer cells by increasing caspase-1, activating GSDMD, promoting the secretion of IL-1β, translocating HMGB1 towards the cytoplasm, and forming membrane pore (249). And all of these events can be inhibited by caspase-1 inhibitors (249).

Dipeptidyl peptidase 8 and dipeptidyl peptidase 9 (DPP8/9) are two members of the dipeptidyl peptidase IV family (250). Recent studies have found that small-molecule DPP8/9 inhibitors can induce pyroptosis in human AML cell lines and primary AML samples by activating the caspase-1/GSDMD pathway, which represents a brand-new therapeutic strategy for AML (250).

2-(anaphthoyl)ethyltrimethylammonium iodide (*α*-NETA), a reversible choline acetylcholine transferase inhibitor, has been reported to induce pyroptosis and inhibit the proliferation of epithelial ovarian cancer cells *in vitro* and *in vivo via* activating caspase-4/GSDMD pathway (251).

In addition to inducing ferroptosis, iron, an essential factor in ROS modulation, can enhance melanoma cell pyroptosis by promoting the cleavage of GSDME, which suggests that iron may be a promising sensitizer for ROS-induced melanoma treatment (252).

#### Chemotherapy Drugs

There are a variety of chemotherapy drugs that have been found to induce pyroptosis by activating GSDME in lung cancer cells, gastric cancer cells, melanoma cell lines, SH-SY5Y neuroblastoma cells, and MeWo cells, including doxorubicin, actinomycin-D, bleomycin, 5-fluorouracil, cisplatin, paclitaxel, topotecan, and etoposide (101, 253–255). What’s more, lobaplatin has been observed to induce pyroptosis in colon cancer cells in a dose-dependent manner by the caspase-3/GSDME pathway (256). Further studies have revealed that lobaplatin induces the elevation of ROS and the phosphorylation of JNK, and activated JNK recruits BCL-2 associated X to the mitochondria and subsequently promotes the release of cytochrome c from the mitochondria to cytosol, being followed by the activation of caspase-3/GSDME-depended pyroptosis (256).

In addition, some chemosensitizers that significantly promote the anti-cancer efficacy of chemotherapy drugs have been implicated in pyroptosis. Recent studies have shown that, in addition to inducing necroptosis in prostate cancer cells, the PLK1 inhibitor BI2536 sensitizes esophageal squamous cell carcinoma cells to cisplatin *in vivo* and *in vitro* by inhibiting the DNA damage repair and promoting pyroptosis (257). Mechanistic studies have demonstrated that the combination of BI2536 and cisplatin can significantly increase the expression of caspase-3 and ensuing cleaved GSDME, which results in the pyroptosis of esophageal squamous cell carcinoma cells (257). What’s more, the silence of GSDME in certain cancer cell types is relieved by decitabine, a kind of DNA methyltransferase inhibitor, which leads to the elevated expression of GSDME and the enhanced sensitivity of GSDME-silenced cancer cells to doxorubicin (101).

#### Molecular Targeted Therapy

Molecular targeted therapy has been applied in cancer management for several decades. Recent studies have revealed that pyroptosis of cancer cells can be induced by molecular targeted agents specifically targeting K-*Ras*-, epidermal growth factor receptor (EGFR)- or anaplastic lymphoma kinase-driven lung cancer (258). Upon molecular targeted agents, such as trametinib, erlotinib, and ceritinib, caspase-3 can be activated and subsequently cleave GSDME, followed by the permeabilized cytoplasmic membrane and pyroptosis induction (258). Similarly, a combination of B-Raf proto-oncogene inhibitors and mitogen-activated protein kinase inhibitors also can induce pyroptosis in melanoma cells by increasing the cleavage of GSDME and the release of HMGB1 (259).

#### Nanomedicine

In addition to the above various compounds, nano-DDS has also been applied to induce pyroptosis in cancer cells, which is similar to its application in ferroptosis mentioned above. Arsenic trioxide nanoparticles have been reported to cause higher expression of GSDME-NT and stronger pyroptosis than free arsenic trioxide in HCC and mice bearing Huh7 xenografts (260).

In general, pyroptosis is emerging as a potent therapeutic target for various cancers. So far, numerous agents have been declared to be associated with pyroptosis in various cancer cells. And all of these agents collectively regulate the cleavage of GSDMD or GSDME, inducing pyroptosis in different pathways. However, there are still a few clinical trials on anti-cancer drugs related to pyroptosis. More efforts still need to be spared in the anti-cancer drug development based on pyroptosis.

## Conclusion and Perspectives

Over several decades, there are four main types of regulated necrosis that have been well studied and involved in various cancers, including necroptosis, ferroptosis, parthanatos, and pyroptosis. So far, several precise cell death signaling pathways have been revealed, and numerous crucial regulators have been found, which form a complex regulatory network in regulated necrosis. Accordingly, diverse novel agents and drugs have been generated to target related molecules and signaling pathways. Interestingly, many existing agents or drugs, for instance, metformin, also exert their anti-cancer effects through or partially through regulating these various modes of regulated necrosis. Specifically, necroptosis-targeted compounds can bypass the signal pathways of apoptosis and induce cell death, which exhibits a remarkable effect on overcoming apoptosis resistance. Furthermore, the development of anti-cancer drugs based on ferroptosis is the most in-depth, while the research of pyroptosis-targeted pharmacotherapy has just emerged recently. Notably, some of these drugs even simultaneously induce two types of regulated necrosis, which indicates the great promise for further research into the crosstalk of different types of regulated necrosis to develop better anti-cancer strategies. What’s more, the clinical transformation of drugs is also an important part of drug development. At present, oral and intravenous injection are commonly used methods of administration and are considered safe. And many clinical trials have evaluated the safety and anti-cancer efficacy of some novel drugs, but studies involving the metabolism and targeting effects of these drugs *in vivo* are still lacking.

Even though the precise underlying mechanisms have not been entirely revealed due to their high complexity, the numerous findings accumulated in the last decade point to clear directions for the next decade. Currently, increasing newly identified regulators of regulated necrosis have been found, such as CXCL1, NRF2, BAP1, and BRCA1/2. However, these molecules have not been well studied in anti-cancer therapy. Thus, it is very promising to develop new anti-cancer drugs by targeting these molecules. Moreover, nano-DDS, an emerging administration method that can greatly enhance the drug targeting property and anti-cancer efficacy, has only been applied to deliver ferroptosis and pyroptosis inducers. Given its superior advantages of targeting ability and permeability effect, it is meaningful to extend this technique to other modes of regulated necrosis. Taken together, the current limitations of cancer therapy are obvious side effects, low targeting efficacy, anti-apoptotic resistance, etc. Therefore, we highlight the necessity to reveal the more detailed signal pathways of regulated necrosis induced by various anti-cancer compounds, and metabolism and targeting effects of these compounds *in vivo*. And these anti-cancer compounds still need further clinical trials for extrapolating to the clinical application. On this basis, the use of regulated necrosis targeted therapy can reduce the burden of drug management, improve the therapeutic effect, and reduce off-target side reactions and drug resistance. A better understanding of regulated necrosis in cancer will pave a broad road for developing new anti-cancer therapies.

## Author Contributions

YZ, JL, and ZF participated in the design of the review. JL, ZF, MM, YY, and YWa drafted the manuscript and made the original figures. YZ critically revised the texts and figures. YWu, YZ, and YD supervised the research, led the discussion. All authors contributed to the article and approved the submitted version.

## Funding

The current work is supported by the National Natural Science Foundation of China (81772562).

## Conflict of Interest

The authors declare that the research was conducted in the absence of any commercial or financial relationships that could be construed as a potential conflict of interest.

## Abbreviations

AIF, apoptosis-inducing factor; AML, acute myeloid leukemia; ATP, adenosine triphosphate; ACSL4, acyl-CoA synthetase long-chain family member 4; AIDS, Acquired Immune Deficiency Syndrome; BSO, Buthionine sulfoximine; cIAP1/2, cellular inhibitor of apoptosis protein 1/2; CN-A, Cotylenin A; CRC, colorectal cancer; CYLD, cylindromatosis; DAMPs, danger-associated molecular patterns; DAPE, 1, 2-Diarachidonoyl-sn-glycero-3-phosphoethanolamine; DHA, Docosahexaenoic acid; DPP4/8/9, dipeptidyl-peptidase-4/8/9; DPT, Deoxypodophyllotoxin; DSBs, double-strand DNA breaks; EGFR, epidermal growth factor receptor; FADD, FAS associated death domain; FLIP_L_, FLICE-like inhibitory protein long isoform; GPX4, GSH peroxidase 4; GSDMD, gasdermin D; GSH, glutathione; HCC, hepatocellular carcinoma; HMGB1, high-mobility group box 1; IFN-*γ*, interferon gamma; IL, interleukin; JNK, c-Jun NH2-terminal kinase; Keap1, Kelch-like ECH-associated protein 1; LPCAT3, lysophosphatidylcholine acyltransferase 3; MIF, migration inhibitory factor; MLKL, mixed lineage kinase domain-like; NAC, N-acetyl-L-cysteine; NAD^+^, oxidized nicotinamide adenine dinucleotide; nano-DDS, nanoparticulate drug delivery systems; NEC1, necrostatin-1; NF-*κ*B, nuclear factor-*κ*B; NOXs, NADPH oxidases; NRF2, nuclear factor erythroid 2-related factor 2; PAMPs, pathogen-associated molecular patterns; PAR, poly (ADP-ribose); PARG, PAR glycohydrolase; PARP1, poly (ADP-ribose) polymerase 1; PDAC, pancreatic ductal adenocarcinoma; PEITC, phenethyl isothiocyanate; PLK1, Polo-like Kinase 1; PUFA, polyunsaturated fatty acid; RCD, regulated cell death; RIPK1/3, receptor-interacting protein kinase 1/3; ROS, reactive oxygen species; RSL3/5, Ras-selective lethal small molecule 3/5; SAT1, spermidine/spermine N1-acetyltransferase; SMAC, second mitochondria-derived activator of caspase; STAT3, signal transducer and activator of transcription 3; TFR1, transferrin receptor 1; TNF, tumor necrosis factor; TRADD, TNF-receptor-associated death domain; TRAF2/5, TNF receptor-associated factor 2/5; 3-AB, 3-aminobenzamide.
